# Necrostatin-1 Attenuates Trauma-Induced Mouse Osteoarthritis and IL-1β Induced Apoptosis via HMGB1/TLR4/SDF-1 in Primary Mouse Chondrocytes

**DOI:** 10.3389/fphar.2018.01378

**Published:** 2018-11-27

**Authors:** Shuang Liang, Zheng-tao Lv, Jia-ming Zhang, Yu-ting Wang, Yong-hui Dong, Zheng-gang Wang, Kun Chen, Peng Cheng, Qing Yang, Feng-jing Guo, Wei-wei Lu, Wen-tao Zhu, An-min Chen

**Affiliations:** ^1^Department of Orthopedics, Tongji Hospital, Tongji Medical College, Huazhong University of Science & Technology, Wuhan, China; ^2^Department of Oral Medicine, Infection and Immunity, Harvard School of Dental Medicine, Boston, MA, United States; ^3^Department of Orthopaedic Surgery, Henan Provincial People’s Hospital, Zhengzhou, China

**Keywords:** necrostatin-1, osteoarthritis, chondrocyte, necroptosis, apoptosis, inflammation

## Abstract

Necrostatin-1 (Nec-1) is a specific small molecule inhibitor of receptor-interacting protein kinase 1 (RIPK1) that specifically inhibits phosphorylation of RIPK1. RIPK1 regulates inflammation and cell death by interacting with receptor-interacting serine/threonine protein kinases 3(RIPK3). We hypothesized that Nec-1 may have anti-inflammatory efficacy in patients with osteoarthritis (OA), as the pathophysiology of OA involves the activation of inflammation-related signaling pathways and apoptosis. In this study, we explored the effects of Nec-1 on interleukin (IL)-1β-induced inflammation in mouse chondrocytes and the destabilised medial meniscus (DMM) mouse model. Inhibiting RIPK1 with Nec-1 dramatically suppressed catabolism both *in vivo* and *in vitro*, but did not inhibit changes in subchondral bone. Nec-1 abolished the *in vitro* increases in matrix metalloproteinase (MMP) and ADAM metallopeptidase with thrombospondin type 1 motif 5 (ADAMTs5) expression induced by IL-1β. However, adding high-mobility group box 1 (HMGB1) partially abrogated this effect, indicating the essential role of HMGB1 and Nec-1 in the protection of primary chondrocytes. Furthermore, Nec-1 decreased the expression of Toll-like receptor 4 (TLR4) and stromal cell-derived factor-1 (SDF-1), and attenuated the interaction between TLR4 and HMGB1. Western blot results suggested that Nec-1 significantly suppressed IL-1β-induced NF-κB transcriptional activity, but not MAPK pathway. Micro-computed tomography, immunohistochemical staining, and Safranin O/Fast Green staining were used *in vivo* to assess the degree of destruction of OA cartilage. The results show that NEC-1 can significantly reduce the degree of destruction of OA cartilage. Therefore, Nec-1 may be a novel therapeutic candidate to treat OA.

## Introduction

Osteoarthritis (OA) is a common, age-related, degenerative disease characterized by the loss of articular cartilage integrity in the joint area, with varying degrees of articular osteophyte formation, subchondral bone remodeling, and synovitis (Glyn-Jones et al., 2015). The main risk factors for OA are age, gender, heredity, joint injuries, and obesity (Prieto-Alhambra et al., 2014). As one of the most common joint diseases, OA affects more than 9.5% of men and 18% of women aged >60 years (Woolf and Pfleger, 2003). It mainly arises in the hands, knees, hips and spine and is characterized by joint pain and loss of function (Dieppe and Lohmander, 2005). At present, treatments for OA are related to structural modifications and improving symptoms, including arthroscopic surgery, autologous cartilage transplantation, micro-fracture, and pharmaceutical drugs. However, the socioeconomic burden of treating OA is steadily rising, reaching 1% of the gross domestic product in developed countries (Hiligsmann et al., 2013).

Receptor interacting serine/threonine kinase 1 (RIPK1), a protein that regulates cell death and inflammation, is ubiquitously expressed in the ovaries, lungs, liver, intestines, limbs, and 25 other tissues (Yue et al., 2014). RIPK1 regulates apoptosis and necroptosis via kinase-dependent and -independent functions, which are essential for cell fate and inflammation (Dannappel et al., 2014; Rickard et al., 2014). Based on the RIP homotypic interaction motifs of RIPK1 and TIR-domain-containing adapter-inducing interferon (TRIF), Toll-like receptor (TLR)3, and TLR4 can also indirectly recruit RIPK1 through TRIF (Zhao et al., 2014). RIPK1 is located downstream of the tumour necrosis factor receptor (TNFR)1 and directly binds to the death domain in TNFR1 (Cook et al., 2014). Micheau et al. (Micheau and Tschopp, 2003; Meylan et al., 2004) showed that TNFR1 recruits RIPK1, adaptor TRADD, adaptor protein TRAF2, and ubiquitin ligases cIAP1 and cIAP2 to form complex I, which activates the mitogen-activated protein kinases (MAPK) signaling pathway and the transcription factor nuclear factor-kappaB (NF-κB) signaling pathway. After forming complex I, TRADD, TRAF2, and RIPK1 associate with FADD and caspase-8, constituting complex II and inducing apoptosis (Micheau and Tschopp, 2003). When caspases are inhibited by the pan-caspase inhibitor zVAD.fmk, necroptosis is executed by RIPK1 and/or RIPK3 (Newton, 2015).

Necrostatin-1 (Nec-1) is a specific small molecule inhibitor of RIPK1 that specifically inhibits phosphorylation of RIPK1 and RIPK1-mediated necroptosis and apoptosis (Degterev et al., 2005). Numerous studies have demonstrated that Nec-1 protects against various disease models *in vivo* and *in vitro*. In the ischaemia-reperfusion injury model after lung transplantation, Nec-1 reduces necroptosis and attenuates ischaemia-reperfusion lung injury by inhibiting the expression of RIPK1/RIPK3/MLKL (Kanou et al., 2018). In the cisplatin-induced nephrotoxicity mouse model, Nec-1 decreases apoptosis as well as proinflammatory cytokines and oxidative stress by inhibiting the activation of the NF-κB signaling pathway (Ning et al., 2018). Based on these results, we hypothesized that Nec-1 would have an anti-inflammatory effect on IL1β-induced chondrocytes and would reduce cartilage loss in a mouse model of OA. Consistent with our assumptions, Nec-1 had a significant protective effect on knee OA in mice. We also explored the specific mechanisms of this drug, including inflammation, apoptosis, and necroptosis.

## Materials and Methods

### Reagents and Antibodies

Nec-1 was purchased from Selleck Chemicals (Houston, TX, United States). Nec-1 was dissolved in DMSO for *in vitro* use, and was dissolved in 5% DMSO, 45% PEG300 and ddH_2_O, in turn. Recombinant murine IL-1β (#211-11B) was obtained from PeproTech (Rocky Hill, NJ, United States). Cell Counting Kit-8 (CCK-8), mouse antibody anti-GAPDH (BM3876), and secondary antibodies were purchased from Boster (Wuhan, China). Antibodies against RIPK1 (#3493), p-ERK (#4370), p-p38 (#4511), p-JNK (#9255), p-IkBa (#2859), p-p65 (#3033) and cleaved caspase-3 (#9964) were purchased from Cell Signaling Technology (Beverly, MA, United States). Rabbit antibodies against ERK (16443-1-AP), p38 (14064-1-AP), JNK (24164-1-AP), IkBa (10268-1-AP), p65 (10745-1-AP), matrix metalloproteinase (MMP)3 (17873-1-AP), TLR4 (19811-1-AP), HMGB1 (10829-1-AP), and stromal cell-derived factor-1 (SDF-1) (17402-1-AP) were purchased from Proteintech Group (Wuhan, Hubei, China). Antibodies against p-MLKL (ab196436), ADAMTs5 (ab41037) and MMP13 (ab39012) were obtained from Abcam (Cambridge, United Kingdom). Mouse SDF-1a/CXCL12 ELISA Kit was purchased from Bangyi (Shanghai, China).

### Animals and the OA Model

Twelve-week-old male C57BL/6 mice (Experimental Animal Centre of Tongji Hospital, Wuhan, China), were housed in a light- and temperature-controlled room and fed a standard diet. The body weight of animals was presented in the Supplementary Table I. The destabilised medial meniscus (DMM) surgical model of OA was produced on the right knee according to a previously published protocol (Glasson et al., 2007). This animal experiment was approved by the Ethics Committee on Animal Experimentation of Tongji Medical College. Forty mice were divided into four groups (*n* = 10 per group): (1) sham group: sham-operated mice administered 15 μL vehicle (5% DMSO, 45% PEG300, and ddH_2_O), (2) the sham + Nec-1 group: sham-operated mice treated with 15 μL Nec-1 (0.0468 mg/Kg), (3) the DMM group: DMM surgery mice administered 15 μL vehicle, and (4) the DMM + Nec-1 group: DMM surgery mice administered 15 μL Nec-1. The Nec-1 solution or vehicle was injected intra-articularly twice weekly for 8 weeks before sacrifice.

### Micro-Computed Tomography (μ-CT) Imaging

The right knee joint structure was analyzed with a μ-CT system (μ-CT Scanco Medical, Bassersdorf, Switzerland). Images were obtained at 100 kV and 98 μA, with the resolution set to 10.5 μm. The three-dimensional images were reconstructed using the software built into the μ-CT system. Bone volume/tissue volume (BV/TV), trabecular thickness (Tb.Th.), trabecular number (Tb.N.) and trabecular separation (Tb.Sp.) were analyzed using the μ-CT system.

### Histological Staining and Analysis

After the animals were euthanised, right knee joint samples were fixed in 4% paraformaldehyde for 48 h, decalcified with 10% EDTA solution for 2 weeks, and embedded in paraffin wax. The knee joints were cut to a 4-μm thickness in the sagittal plane. Ten consecutive sections out of 25 were selected to represent the weight-bearing area of the femur and tibia, respectively, and were selected for scoring and quantifying IHC. The structure of the knee joints was observed under Safranin O/Fast Green staining, and the severity of OA changes was assessed using the Osteoarthritis Research Society International (OARSI) histopathology scoring system (Glasson et al., 2010). The DAB Histostain-SP kit was used for immunohistochemistry. Sections were incubated with specific antibodies to observe expression changes in MMP3, MMP13, and ADAMTs5. The number of positively stained cells was counted in the articular cartilage under a digital microscope (Nikon ECLIPSE Ti-S, Nikon, Tokyo, Japan).

### Cell Culture

Mouse primary chondrocytes were collected from the knee cartilage of new-born C57BL/6 mice as described previously (Li et al., 2018). Articular cartilage derived from the knee joints was dissected into pieces in sterilized phosphate-buffered saline (PBS) and digested with 0.25% trypsin at 37°C for 30 min. The pieces were collected and incubated with 0.25% collagenase II at 37°C for 6 h. The primary chondrocytes were resuspended and cultured in DMEM/F12 with 10% foetal bovine serum (FBS), 100 U/mL penicillin and 100 mg/mL streptomycin sulfate in 25 cm^2^ flasks at 37°C under 5% CO_2_. Chondrocytes that had been passaged one or two times were used for the experiments.

### Cell Viability Assay

The CCK-8 assay (Boster) was used to assess cell proliferation and viability. Briefly, primary chondrocytes were seeded at a density of 10,000 cells per well in 96-well plates. The next day, culture medium containing DMSO (vehicle) or an equal volume of Nec-1 (30 μmol/L) was added every day. The culture medium was replaced after 24, 48, and 72 h with 100 μL of medium containing a 10% CCK-8 solution, then incubated for 1 h in the dark at 37°C. Absorbance was measured at 450 nm with an ELX800 microplate reader (Bio-Tek, Winooski, VT, United States).

### Apoptosis Analysis

An Annexin V-FITC/propidium iodide (PI) kit was used to detect apoptosis in chondrocytes in the presence of IL-1β (5 ng/mL) with or without Nec-1 (30 μmol/L). The chondrocytes were collected after treatment, washed three times in ice-cold PBS and resuspended in binding buffer. Then, 5 μL PI and 5 μL Annexin V were added to the buffer for 15 min at 4°C in the dark. A FACS Calibur flow cytometer (BD, Franklin Lakes, NJ, United States) was used to analyze the apoptotic cells in the early and late phases according to the manufacturer’s instructions.

### Immunofluorescence

Primary chondrocytes were fixed in 4% paraformaldehyde for 15 min at room temperature, permeabilised with 0.1% Triton X-100 for 5 min and blocked with 0.1% bovine serum albumin for 30 min. The cells were incubated with primary antibody against HMGB1 overnight at 4°C, followed by goat anti-rabbit IgG/Cy3 (Invitrogen, Camarillo, CA, United States) as a second antibody for 1 h at room temperature. Finally, 4,6-diamidino-2-phenylindole (DAPI) was added to the cells to stain the nuclei in the dark. Immunofluorescence was imaged with a fluorescence microscope (Evos Flauto; Life Technologies, Carlsbad, CA, United States).

### Co-immunoprecipitation and Western Blotting

IgG and Protein A+G agarose were added to the cell lysates for 2 h to block non-specific binding. A primary antibody was added to the mixture with shaking overnight at 4°C, followed by another incubation with Protein A+G agarose for 3 h. After centrifugation, the Protein A+G agarose was washed five times with lysis buffer, resuspended in sodium dodecyl sulfate sample buffer and boiled for 10 min. The BCA assay (Boster) was used to measure the concentration of protein in the samples used for western blotting. Equivalent quantities of samples were subjected to sodium dodecyl sulfate-polyacrylamide gel electrophoresis and then transferred to polyvinylidene fluoride membranes (Millipore, Billerica, MA, United States). After incubation with primary antibodies overnight at 4°C on a shaker, the membranes were incubated with secondary antibodies and then scanned with the ChemiDocTM XRS C System (Bio-Rad Laboratories, Hercules, CA, United States).

### Statistical Analysis

All experiments were independently performed at least three times with similar results. The results are presented as the mean ± standard deviation. Student’s *t-*test was used to assess differences between two groups, and one-way analysis of variance (ANOVA) followed by Dunnett’s *post hoc* test was used to compare groups. *P*-values < 0.05 were considered significant.

## Results

### Nec-1 Attenuates Cartilage Degeneration *in vivo*

**FIGURE 1:**
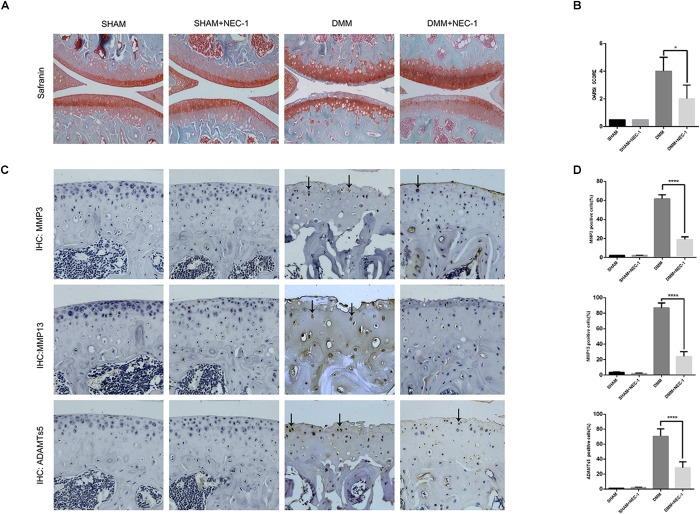
Inhibition of RIPK1 with Nec-1 attenuates cartilage degeneration. **(A)** Safranin O/Fast Green staining in the sagittal plane of tibial and femoral cartilage from the sham, sham + Nec-1, destabilised medial meniscus (DMM), and the DMM + Nec-1 groups (*n* = 10). Scale bar = 100 μm. **(B)** Osteoarthritis Research Society International (OARSI) histopathology scores of sham and DMM mice treated with vehicle and Nec-1, respectively. **(C)** Expression of MMPs and ADAMTs5 were determined by immunohistochemical staining of the cartilage from each group (*n* = 10). **(D)** MMPs and ADAMTs5 positive cells were quantified using Image-J software. ^∗^*P* < 0.05, ^∗∗∗∗^*P* < 0.0001.

We confirmed a significant change in tibial and femoral cartilage in DMM mice compared with sham-operated mice. Specifically, Safranin O/Fast Green staining demonstrated the structural changes, fibrillations, and vertical clefts of cartilage in the DMM mice (Figure 1A). MMP3, MMP13, and ADAMTs5 were expressed in articular cartilage according to the immunohistochemical staining (Figures 1C,D). Using the OARSI scores system, the degree of OA cartilage destruction in these four groups was quantified by three blinded observers (YW, JZ, and ZW). The OARSI scores (Figure 1B) were significantly lower (tibia: 2 points; femur: 2 points; *P* < 0.05) in the DMM + Nec-1-treated group than those in the DMM-treated mice (tibia: 4 points; femur: 4 points). No significant differences were found between the Sham + Nec-1-treated group and the sham controls (*P* > 0.05). These results indicate that Nec-1 attenuated the destruction of articular cartilage in DMM mice, and it had similar effects on decreasing the expression of MMP3, MMP13, and ADAMTs5 in articular cartilage compared to the sham group. The OARSI scores revealed the significant protective effect of Nec-1 against OA in mice.

### Nec-1 Does Not Affect Tibial Subchondral Bone Remodeling

**FIGURE 2:**
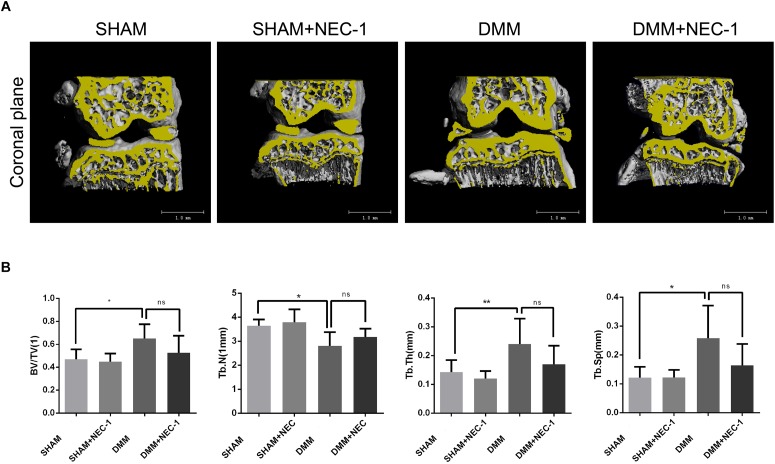
Inhibition of RIPK1 with Nec-1 does not affect tibial subchondral bone remodeling. **(A)** Three-dimensional micro-computed tomography coronal views of the medial and lateral tibial subchondral bone from each group. **(B)** Histograms represent the trabecular structural analysis of tibia platform subchondral trabecular: trabecular bone volume/tissue volume (BV/TV), trabecular number (Tb.N), trabecular thickness (Tb.Th) and trabecular separation (Tb.Sp). Data are the mean ± SD. *n* = 10. DMM different from sham (^∗^*P* < 0.05, ^∗∗^*P* < 0.01); ns: not significant.

The effects of Nec-1 on the structure of tibial subchondral bone were measured and analyzed by μCT in the DMM OA mice. BV/TV, Tb.Th, and Tb.Sp increased significantly in the DMM group compared with the sham group, whereas Tb.N decreased (Figures 2A,B). However, no differences in BV/TV, Tb.Th, Tb.Sp, or Tb.N were observed between the DMM + Nec-1 and sham groups. These results indicate that Nec-1 does not affect tibial subchondral bone remodeling in mice with OA.

### Nec-1 Inhibits IL-1β-Induced Catabolism *in vitro*

**FIGURE 3:**
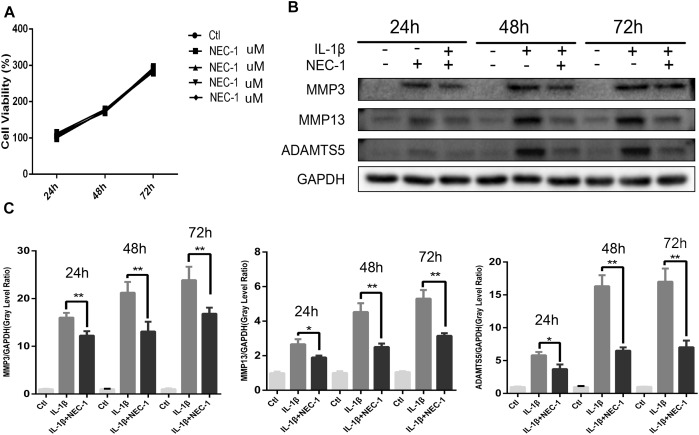
Effects of Nec-1 on interleukin (IL)-1β-induced matrix metalloproteinase (MMP) and ADAM metallopeptidase with thrombospondin type 1 motif 5 (ADAMTS5) expression. Primary chondrocytes pretreated with Nec-1 (30 μmol/L) for 30 min were treated with IL-1β (5 ng/mL) for further 24, 48, and 72 h. **(A)** Cells were treated with Nec-1 (30 μmol/L) for 24, 48, and 72 h, and viability was evaluated by the Cell Counting Kit-8 (CCK-8) assay. **(B)** The protein levels of MMP-3, MMP-13, and ADAMTs5 were determined by western blot analysis. **(C)** Relative protein expression was quantified using ImageJ software. The experiments were repeated three times independently. ^∗^*P* < 0.05, ^∗∗^*P* < 0.01 vs. IL-1β group.

The CCK-8 assay was performed to measure the cytotoxic effects of Nec-1 on chondrocytes. As shown in Figure 3A, Nec-1(30 μmol/L) did not decrease cell viability or proliferation after 24, 48, or 72 h. MMPs and ADAMTs are key regulators of cartilage destruction (Mengshol et al., 2000; Liang et al., 2018). To determine the anti-inflammation significance of Nec-1 on IL-1 induced release of catabolic enzymes by chondrocytes, the cells were co-treated with Nec-1 in the presence or absence of IL-1β for 48 h. As shown in Figures 3B,C, the protein levels of MMP3, MMP13, and ADAMTs5 increased significantly after induction by IL-1β, but were significantly reversed by Nec-1 treatment in 48 and 72 h.

### Nec-1 Prevents Chondrocyte Inflammation by RIPK1/HMGB1/TLR4 Signaling and Apoptosis but Not Necroptosis

**FIGURE 4:**
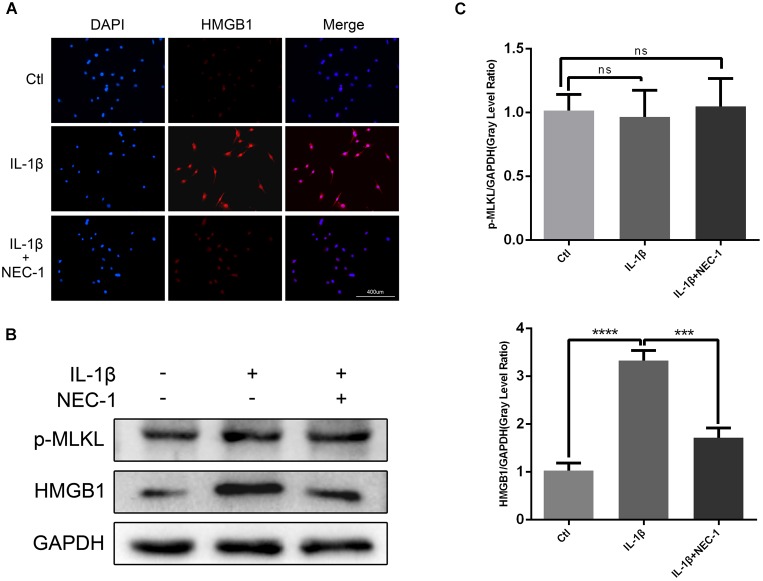
Nec-1 acts through high-mobility group box 1 (HMGB1) to regulate chondrocytes. **(A)** The subcellular location of HMGB1 was determined by immunofluorescence staining for endogenous HMGB1 (red) and DAPI for nuclei (blue). Bar = 400 μm. **(B)** The protein levels of HMGB1 and p-MLKL were determined by western blot analysis. **(C)** Relative protein expression was quantified using ImageJ software. The experiments were repeated three times independently. ^∗∗∗^*P* < 0.0006, ^∗∗∗∗^*P* < 0.0001.

**FIGURE 5:**
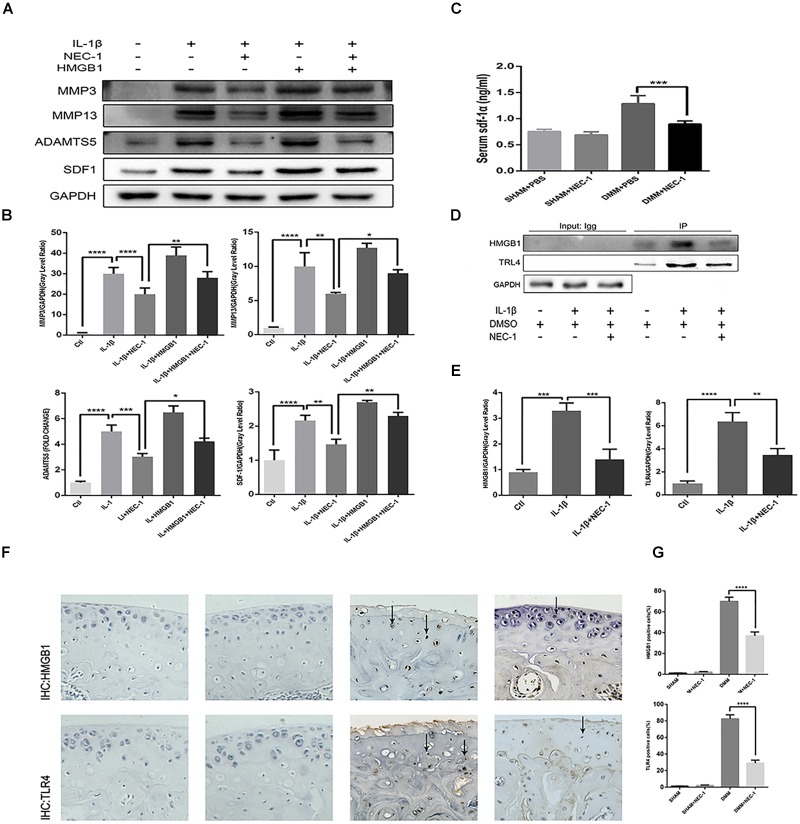
The RIPK1/toll-like receptor 4 (TLR4) pathway attenuates chondrocyte degeneration induced by Nec-1. **(A,B)** The expression levels of MMPs, ADAMTs5, and stromal cell-derived factor-1 (SDF1) in primary chondrocytes from the ctl, IL-1β, IL-1β + Nec-1, IL-1β + HMGB1, and IL-1β + HMGB1 + Nec-1 groups were determined by western blotting. **(C)** Serum levels of SDF-1α protein were measured by ELISA. Serum were collected from mice euthanized at 8 weeks post-DMM surgery (*n* = 10 for each group). **(D,E)** Interactions between HMGB1 and TLR4 were detected by co-immunoprecipitation. Relative protein expression was quantified using ImageJ software. The relative protein expression levels of HMGB1 and TLR4 were normalized to GAPDH and expressed as a relative value. **(F,G)** Expression of HMGB1 and TLR4 were determined by immunohistochemical staining of the cartilage from each group (*n* = 10). HMGB1 and TLR4 positive cells were quantified using Image-J software. The experiments were repeated three times independently. ^∗^*P* < 0.05, ^∗∗^*P* < 0.01, ^∗∗∗^*P* < 0.0006, and ^∗∗∗∗^*P* < 0.0001.

HMGB1, a key mediator of inflammation, plays an important role in initiating OA and rheumatoid arthritis (Wang et al., 2015; Jiang et al., 2017). In our experiment, the HMGB1 expression level decreased significantly in the IL-1β + Nec-1 group compared to the IL-1β group (Figures 4B,C). The immunofluorescence analysis showed similar results. IL-1β promoted HMGB1 expression and translocation from the nucleus to the cytoplasm, and these effects were significantly inhibited by Nec-1 (Figure 4A). To determine the role of HMGB1 in attenuated inflammation, we treated chondrocytes with rHmgb1 (HMGBiotech, HM-115) and IL-1β. The results showed that the decreased expression of MMP3, MMP13, ADAMTs5, and SDF1 was abrogated by adding HMGB1 (Figures 5A,B). The tendency is consistent with the results of the cell experiments. As the interaction between HMGB1 and TLR4 is responsible for amplifying the inflammatory response, co-immunoprecipitation was performed with HMGB1 and TLR4 (Figures 5D,E). The results indicated that Nec-1 decreased the interaction between HMGB1 and TLR4 induced by IL-1β.

**FIGURE 6:**
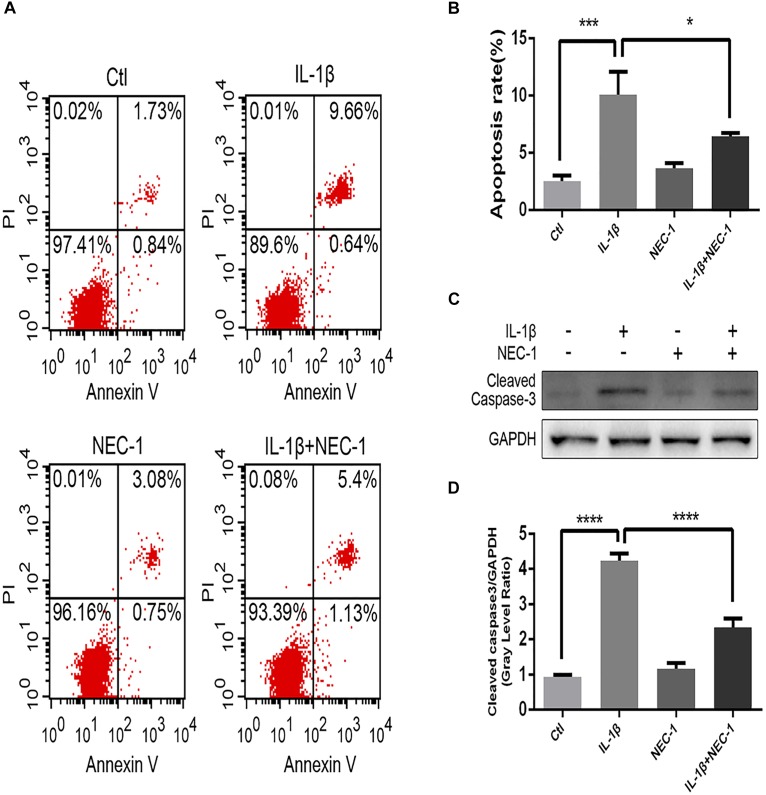
Nec-1 protects against IL-1β-induced apoptosis. **(A)** Chondrocytes were stained with Annexin V-FITC/propidium iodide (PI) and detected by flow cytometry. **(B)** Histograms represent the rate of apoptosis. Ctl: 2.57%; IL-1β: 10.3%; Nec-1: 3.83%; IL-1β + Nec-1: 6.53%. **(C,D)** The cleaved caspase-3 protein level was determined by western blot analysis. The experiments were repeated three times independently. ^∗^*P* < 0.05, ^∗∗∗^*P* < 0.0006, and ^∗∗∗∗^*P* < 0.0001.

The levels of apoptosis and expression of cleaved caspase-3 were determined to investigate the role of apoptosis in the anti-inflammatory effects of Nec-1 in articular chondrocytes. The expression of cleaved caspase-3, the key major effector of caspases, was significantly augmented in IL-1β-induced chondrocytes, which was reversed by Nec-1 (Figures 6C,D). Correspondingly, apoptosis was detected by Annexin V-FITC/PI staining and the levels were lower in the IL-1β-Nec-1-combined treatment group (Figures 6A,B), in agreement with the changes in the amount of cleaved caspase-3. There were no differences in necroptosis marker P-MLKL in our experiment (Figures 4B,C). These results demonstrate that Nec-1 protects chondrocytes from inflammation by inhibiting RIPK1/HMGB1/TLR4 signaling and apoptosis, but not necroptosis.

### Nec-1 Reduces Inflammatory Markers *in vivo*

The role of SDF-1in the pathogenesis of OA has attracted more attention in recent years. In our *in vivo* experiment, ELISA indicated serum SDF-1 expression in DMM mice and sham mice. The levels of serum SDF-1α decreased by 30.5% in DMM + Nec-1 group at 8 weeks post-surgery compared with DMM group; this difference was statistically significant (Figure 5C). The tendency is consistent with the results of the cell experiments. The effects of Nec-1 on the expression of HMGB1 and TLR4 in the cartilage were also validated by ICH in the mouse model (Figures 5F,G). The results showed that Nec-1 significantly decreased the expression of HMGB1 and TLR4 in the cartilage of DMM + Nec-1 group compared with DMM group.

### HNF-κB but Not MAPK Is Involved in the Protective Effect of Nec-1 in Chondrocytes

**FIGURE 7:**
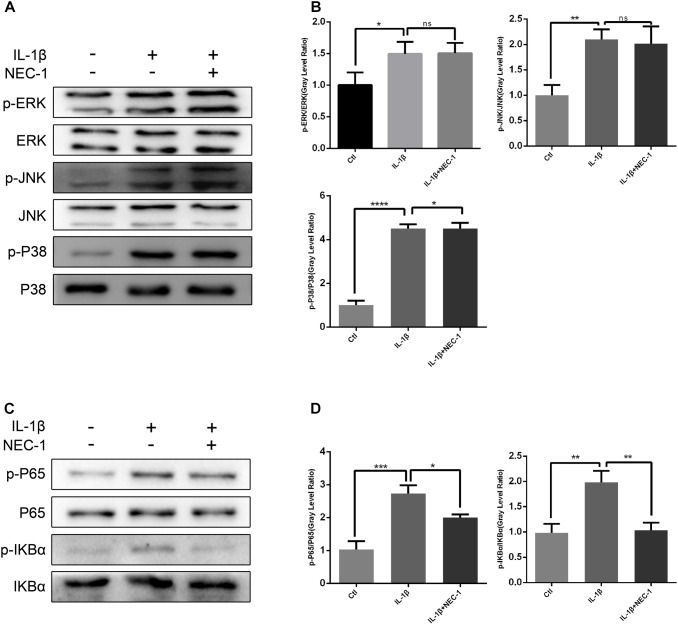
Nec-1 represses activation of the NF-κB signaling pathway in chondrocytes induced by IL-1β. In order to reduce the levels of phosphorylated MAPK and NF-KB, the chondrocytes were serum-starved for 8 h. Since phosphorylated MAPK and NF-KB levels reach a maximum within 30 min and degrade within 1 h, the chondrocytes were treated with IL-1β for 15 min. **(A,B)** Nec-1 did not repress the MAPK signaling pathway. Chondrocytes were starved with DMEM in the absence of FBS for 8 h, and then pre-treated with or without Nec-1 (30 μmol/L) for 1 h. Subsequently, the chondrocytes were treated with IL-1β (5 ng/mL) for 15 min. **(C,D)** Nec-1 represses the NF-κB signaling pathway. The experiments were repeated three times independently. ^∗^*P* < 0.05, ^∗∗^*P* < 0.01, ^∗∗∗^*P* < 0.0006, and ^∗∗∗∗^*P* < 0.0001.

To investigate the exact mechanism of the protective effect of Nec-1 in articular chondrocytes, we checked the activation status of the NF-κB and MAPK pathways in chondrocytes induced by IL-1β combined with/without Nec-1 for 15 min. As shown in Figure 7, Nec-1 significantly inhibited the phosphorylation of IkBa and p65 (Figures 7C,D). No significant differences in the level of phosphorylation of the MAPK pathway were detected between the IL-1β + Nec-1 and control groups (Figures 7A,B).

## Discussion

osteoarthritis is the most common form of arthritis and involves multiple proinflammatory cytokines, including IL-1β, TNF, and IL-6. IL-1β is secreted by chondrocytes, mononuclear cells, osteoblasts, and synoviocytes. It suppresses the production of collagen II and aggrecan and stimulates the release of MMP-3, MMP13, and ADAMTs5. MMPs and ADAMTs are key regulators of cartilage destruction (Lv et al., 2017). Nec-1 is a specific small molecule inhibitor of RIPK1 that specifically inhibits phosphorylation of RIPK1. Although numerous studies have demonstrated that Nec-1 attenuates various *in vivo* and *in vitro* disease models (Zhang et al., 2017), the effects of Nec-1 on mice with OA are unknown. Our study demonstrated that Nec-1 attenuated OA *in vivo* and *in vitro* through the RIPK1/HMGB1/TLR4 and apoptosis pathways. We constructed a destabilized OA animal model by transecting the anterior cruciate ligament of the right knee in mice. After 8 weeks of DMM, we observed significant destruction of articular cartilage and subchondral bone sclerosis in the operated knees.

HMGB1 is a member of the damage-associated molecular patterns (DAMPs) that is released from dead/damaged cells during inflammation, tissue injury, necrosis, and hypoxia (Srikrishna and Freeze, 2009), creating a sustained inflammatory niche. Immunohistochemistry results indicate that the expression of HMGB1 increases in a complete Freund’s adjuvant-induced OA animal model (Li et al., 2016). TLR4, a member of the toll-like receptor family, activates the inflammatory response by interacting with DAMPs (Ooboshi and Shichita, 2016). A previous study demonstrated that HMGB1 is unable to aggravate the inflammatory response in TLR4 knockout mice (Nadatani et al., 2013). Duprez et al. (2011) showed that pretreatment with a RIPK1 inhibitor confers protection against systemic inflammatory response syndrome by reducing the expression of DAMPs. In the present study, we demonstrated that Nec-1 enhanced articular structural recovery by inhibiting the expression of HMGB1 and its interaction with TLR4. The effects of Nec-1 on the expression of HMGB1 and TLR4 in the articular cartilage were validated by ICH in the mouse model. We also explored the role of SDF-1α in the protection of Nec-1. SDF-1α is a catabolic factor that degrades cartilage by decreasing proteoglycan content and increasing MMP activities (Dong et al., 2016). We found that decreased expression of SDF1 was abrogated by adding HMGB1. Our *in vivo* experiment also demonstrated that the levels of serum SDF-1α obviously decreased in DMM + Nec-1 mice compared with DMM mice. Consistent with our findings, Yun et al. (2017) also showed that SDF-1α expression and release increased in HMGB1-stimulated eye inflammation in mice (Yun et al., 2017).

Apoptosis is a type of programmed, highly regulated cell death that includes chromosome condensation, DNA fragmentation, cell shrinkage, plasma membrane blebbing, and the formation of apoptotic bodies (Kerr et al., 1972). Numerous studies have revealed that apoptosis is highly correlated with the degree of cartilage destruction and matrix catabolism in OA tissues (Musumeci et al., 2015). Furthermore, the release of cellular contents and inflammatory mediators from apoptotic cells may play an additional role in aggravating OA (Hwang and Kim, 2015). Chondrocyte degradation and apoptosis may form a vicious cycle. Cleaved caspase-3, the most important executioner caspase, is a specific hallmark of apoptosis. Our *in vitro* study indicated that Nec-1 significantly inhibited apoptosis and inflammation in chondrocytes induced by IL-1β. Necroptosis is a novel mechanism of programmed necrosis that incorporates features of apoptosis and necrosis. Phosphorylation of MLKL by the autophosphorylation of RIPK3 during necroptosis results in the translocation of MLKL from the cytosol to the plasma membrane (Sun et al., 2012). However, our experiments showed that IL-1β was unable to induce necroptosis *in vitro*. A previous study showed that TNF-α and IFN-β cannot induce necroptosis in the absence of zVAD (McComb et al., 2014), which may account for our results.

Some studies have suggested that negative regulation of the NF-κB and MAPK signaling pathways controls RIPK1. NF-κB and MAPK are vital signaling pathways involved in the production of MMPs and the regulation of chondrocyte proliferation, apoptosis and differentiation. Our results show that Nec-1 inhibited the phosphorylation of IkB-α and p65, suggesting a possible underlying mechanism for the inhibitory effects of Nec-1 in OA. However, changes in the phosphorylation levels of JNK, ERK and p38 were not significant, and were inconsistent with previous studies (Kemeny et al., 1988; Kim and Lee, 2017).

As the most important proinflammatory cytokine, IL-1β contributes to the pathogenesis of OA through several mechanisms including downregulation of anabolic events and upregulation of catabolic and inflammatory responses (Kapoor et al., 2011). Nowadays, IL-1β stimulation (2–20 ng/ml) is used as a conventional *in vitro* model to recapitulate the pathological condition of DMM OA model (Wang et al., 2017; Hu et al., 2018; Tan et al., 2018). Yasuhito (Song et al., 2018) and Shinsuke (Kihara et al., 2017) have demonstrated that IL-1β-positive chondrocytes increased significantly in DMM mice compared with levels observed in sham mice. It is well known that estrogen plays an important role in the progression of OA (Ma et al., 2007; Tanamas et al., 2011). OA is more prevalent in men than women before age 50. In order to rule out the effects of estrogen in mice model, only male mice were chosen in our experiments. The DMM mouse model used in our study is advantageous as it results in mild OA and is more sensitive to the effects of treatment, compared with other destabilized OA animal models. This DMM model better simulates the disease modifications of aged spontaneous mouse models of OA (Glasson et al., 2007). In other cell line experiments, the dosage of Nec-1 used in cell culture models was 10 to 300 μM (Zheng et al., 2014; Chen et al., 2015; Suzuki et al., 2015; Wu et al., 2015), and 1.65–30 mg/kg in animal models (Najjar et al., 2016; Ning et al., 2018). In our study, the dosage of Nec-1 used in animal was 0.0468 mg/Kg body weight, and 30 μM in cell culture models. In conclusion, Nec-1 had a protective effect against the destabilized OA animal model and chondrocyte inflammation. This protective effect was attributed to inhibiting apoptosis, HMGB1 expression, and the NF-κB signaling pathway. Furthermore, the mechanisms associated with HMGB1 included interactions with TLR4 and the suppression of SDF1. However, further studies are needed to investigate other Nec-1 targets and the details of the role of RIPK1 in the pathology of OA.

## Author Contributions

A-mC and W-tZ designed the experiments and revised the manuscript. SL conducted the experiments and prepared the manuscript. Z-tL and J-mZ analyzed the data. Y-tW, Z-gW, KC, W-wL, and PC contributed to clarifying and supervising the writing of the manuscript. F-jG and QY helped with manuscript revision. All authors have read and approved the final manuscript.

## Conflict of Interest Statement

The authors declare that the research was conducted in the absence of any commercial or financial relationships that could be construed as a potential conflict of interest.
