# Supplementary material for: Necrostatin-1 Attenuates Trauma-Induced Mouse Osteoarthritis and IL-1β Induced Apoptosis via HMGB1/TLR4/SDF-1 in Primary Mouse Chondrocytes
Source: Front Pharmacol. 2018 Nov 27;9:1378. doi: 10.3389/fphar.2018.01378 (PMC6277802; doi:10.3389/fphar.2018.01378)
Supplement: Supplementary file 1 [file Table_1.DOCX]

**Supplementary Table I**

Weight change in mice before and after knee joint instability surgery. Values are means±SD. Differences between mice, at the surgery or sacrifice time point, were assessed using a KruskaleWallis with Dunn’s post-test. There was no statistical difference between groups.

| group | n | body weight (g) | |
| --- | --- | --- | --- |
|  |  | At surgery | At sacrifice |
| SHAM+PBS | 10 | 24.7±0.75 | 26.9±0.67 |
| SHAM+NEC-1 | 10 | 25.0±0.81 | 27.5±0.81 |
| DMM+PBS | 10 | 25.0±0.48 | 27.5±0.48 |
| DMM+NEC-1 | 10 | 25.3±0.56 | 28.0±0.56 |
